# HAVEN: Haptic And Visual Environment Navigation by a Shape-Changing Mobile Robot with Multimodal Perception

**DOI:** 10.1038/s41598-024-75607-7

**Published:** 2024-11-06

**Authors:** Barry W. Mulvey, Thrishantha Nanayakkara

**Affiliations:** Dyson School of Design Engineering, Imperial College London, London, SW7 2DB UK

**Keywords:** Embodied intelligence, Deformable robot, Bio-inspired robotics, Multimodal perception, Mechanical engineering, Electrical and electronic engineering

## Abstract

Many animals exhibit agile mobility in obstructed environments due to their ability to tune their bodies to negotiate and manipulate obstacles and apertures. Most mobile robots are rigid structures and avoid obstacles where possible. In this work, we introduce a new framework named Haptic And Visual Environment Navigation (HAVEN) Architecture to combine vision and proprioception for a deformable mobile robot to be more agile in obstructed environments. The algorithms enable the robot to be autonomously (a) predictive by analysing visual feedback from the environment and preparing its body accordingly, (b) reactive by responding to proprioceptive feedback, and (c) active by manipulating obstacles and gap sizes using its deformable body. The robot was tested approaching differently sized apertures in obstructed environments ranging from greater than its shape to smaller than its narrowest possible size. The experiments involved multiple obstacles with different physical properties. The results show higher navigation success rates and an average 32% navigation time reduction when the robot actively manipulates obstacles using its shape-changing body.

## Introduction

With the emergence of new robot-based applications, robots have to plan and execute tasks in the presence of environmental uncertainties which makes sensing an important component. Robots must be equipped with different sensing modalities to be able to operate in unstructured environments. The combination of different modalities can have a synergistic effect, including cases in which the use of a single sensor modality may fail^1,2^. It has been shown that the fusion of different sensing modalities (including tactile sensing) can increase successful execution of tasks requiring interaction with the environment^3,4^.

**Fig. 1:**
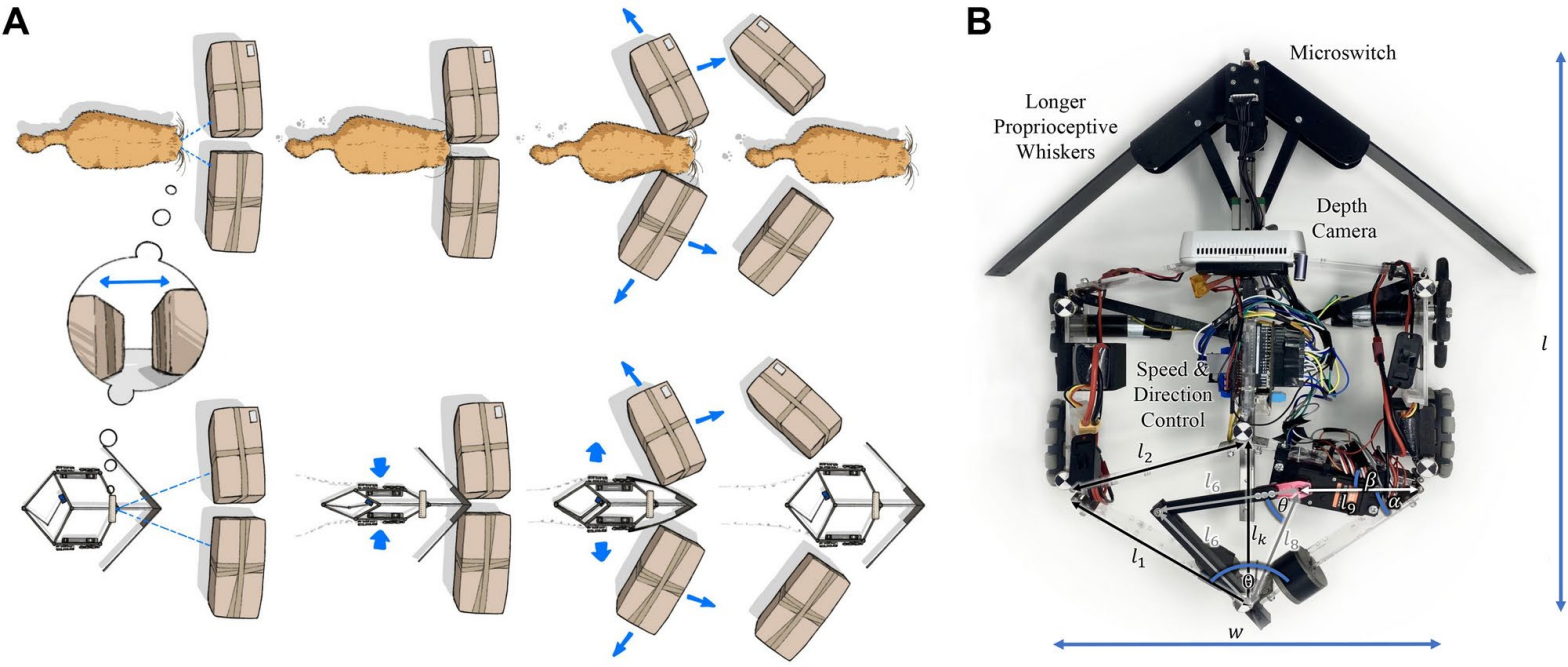
(**A**) Taking a bio-inspired approach from animals such as cats, we present a robot which uses visual and proprioceptive sensing to be predictive, reactive, and active in its locomotion and interactions. From left-right: The robot analyses its environment and observes the small gap between the obstacles, approaches at its narrowest body shape, expands its shape to actively widen the gap, and is able to resume its natural shape once it has progressed. (**B**) Overhead view of the augmented deformable mobile robot DeforMoBot detailing design measurements and augmentations including depth camera, longer whiskers, micro switch, and speed and direction control.

As tactile sensing performance improves, sensor fusion becomes more important in multiple applications^5^. However, in the literature on haptics, exploration has been treated only to a limited extent compared to grasping^6^. The majority of mobile robot path planning methods focus on obstacle avoidance, rather than object traversal or manipulation.

Deformable or transformable robots offer the ability to change shape depending on their task or requirement. Some examples include continuum^7,8^, origami^9,10^, and modular robots^11,12^. The development of deformable robots is important and useful because they can adapt their bodies to suit their environments, navigate more efficiently and safely, and present a wide range of potential applications. Some of these applications include medical and healthcare^13,14^, household^15,16^, search and rescue^17,18^, and environmental and space exploration^19–21^. Deformability or compliance in robots is also advantageous since unexpected collisions can be somewhat tolerated^22^, similar to how animals minimise collision force to reduce risk of injury. Other benefits of shape change include variable stability and centre of mass (which could be extended for advantages with payload capability), changeable points of contact with the ground (which can enable the robot to drive on more desirable surfaces or avoid potential hazards), varying turning circle/radius (especially useful in cluttered or obstructed environments), and inherent damage resilience and mechanical robustness (increasing ability to absorb impacts or adapt in case of component failure)^23^.

We previously developed DeforMoBot^24^, a deformable mobile robot capable of using proprioceptive whisker feedback to adjust its shape in real time in order to traverse obstacles in its path. The robot’s design is bio-inspired by animals such as cats which have the ability to squeeze through spaces narrower than their normal body size.

In this work, we propose a new framework named Haptic And Visual Environment Navigation (HAVEN) Architecture. This architecture enables the augmented robot to autonomously choose between *reactive* traversal and *active* mobile manipulation of obstacles based on a visual prediction algorithm. The IEEE Robotics and Automation Society’s Technical Committee for Mobile Manipulation defines mobile manipulation as robotic tasks that require a synergistic combination of navigation and interaction with the environment^25^. Specifically, using vision and proprioception algorithms, the robot is now

- Predictive, by visually analysing the environment and preparing its body for the given circumstances;
- Reactive, by responding to proprioceptive feedback; and
- Active, by manipulating obstacles and gap sizes.

While most robots do not engage tasks through kinematic tuning such as stiffness and shape change, our robot can take action both in state space and through kinematic tuning, taking inspiration from biological counterparts. The motion planning problem of Navigation Among Movable Obstacles explores the potential for obstacle manipulation^26^, but has not been thoroughly explored in unstructured environments.

Many animals (such as cats, rats, rabbits, and seals) use a combination of their eyes and whiskers for perception of their environment. Likewise, we have enabled the robot to benefit from multimodal perception, as shown in Fig. 1. Building on proprioceptive sensing developed previously^24^, the robot can now use vision to analyse its environment to find the properties and location of the biggest gap in the scene and prepare itself accordingly (through speed and shape change). Real-time proprioceptive feedback can then allow the robot to update its shape in the course of its navigation and also assists with edge cases. The robot can also manipulate obstacles and gaps through active shape change.

The remainder of the paper is organised as follows. “Related work” discusses relevant related work. “Method” presents the methodology including algorithms and the proposed HAVEN Architecture. “Experiments and results” explains the experimentation and also analyses the results obtained. Finally, “Conclusions” presents the conclusions of this work.

## Related work

In general, a major aim of robot navigation is to avoid collisions between the robot and its environment^27,28^. However, this is limiting, especially in obstructed environments, where obstacles can be moved or manipulated to facilitate the robot’s navigation through the environment. This is evidenced by the fact that in many works to date that have focused on outdoor locomotion, navigation has been limited to uneven terrain^29–31^. To enable more robust navigation in the face of these challenges, the integration of further sensing such as proprioception can be beneficial.

The effective combination of visual and tactile modalities remains a challenging problem^32^. The fusion of vision and haptics in robotics has predominantly been utilised in object grasping, and has also been used in virtual reality applications. Using vision alone, robots cannot infer physical properties of obstacles (beyond geometry, colour, and semantics). Obtaining increased knowledge about its surroundings can help a robot to achieve its goal in unstructured environments. Some work has demonstrated that fusion of visual and tactile sensor data can enable robots to build a more detailed model of objects with which they have interacted. For example, a robot arm with RGB-D camera for tabletop scanning and mapping was able to determine rigidity by poking with force sensing, measure local material type using single-pixel spectroscopy, and predict force distributions by pushing^33^. The combination of tactile sensing and vision for haptic mapping by a robot arm has also been explored where visually similar scenes are assumed to have similar tactile characteristics^34^.

In the context of navigation, the fusion of visual and tactile information could allow a robot to navigate in poor visibility conditions with high occlusion or navigate around objects which are difficult to perceive. At present, there are few works that have fused visual and tactile information for navigation. Some work has been done on this combination for simultaneous localization and mapping (SLAM), examples of which include bio-inspired visuo-tactile SLAM for environment exploration and interaction^35^, and whisker-based tactile SLAM to generate occupancy maps^36^. In other work, a mobile robot used a camera to measure the compression of a passive foam rod which deformed when in contact with objects in its environment^37^. However, these works did not investigate this visual-tactile fusion for mobile manipulation.

The field of mobile manipulation largely consists of robots using manipulator arms to achieve tasks^38,39^. Robot arms can be useful and helpful, but they require additional hardware, computation, and power. Moreover, a manipulator adds an occasionally-used payload and an undesirable kinematic augmentation on the robot. A deformable robot can instead use its own shape-changing body to its advantage and directly interact with and manipulate obstacles.

Affordances can be described as key attributes of an environment which could be perceived by a robot to effectively interact with objects^40^. One particular robot used 3D range sensing and learned to perceive traversability affordances, although the authors reported this sensing as slow, requiring considerable learning data and limiting the robot’s perception and reactivity^41^. Morphology should also be considered for increased agility and robustness^42^ and for understanding affordance detection in organisms and robots. We recently developed the PaTS-Wheel, a passively-transformable single-part wheel that can render hooks when presented with obstacles or uneven terrain^43^. In other recent work, a simulation involving voxel-based soft robots predicted whether they could fit through an aperture using minimal tactile feedback^44^.

Shape-changing robots offer potential beyond the capabilities of rigid robots. Some soft robots have been manufactured for navigating narrow gaps. One such example is a multiple-gait soft robot capable of squeezing into confined spaces^45^. In other work, an inch-long soft robot mimicked the crawling locomotion of a caterpillar with a travelling-wave deformation to squeeze through a narrow slit^46^. However, soft robots have limited force capability^47^. Bio-inspired origami robots combine smart material actuators with folding processes to obtain compliance and soft-body features^9^. Diverse transformations aim to achieve increased mobility and versatility^48^ as well as reconfigurable modularity^49^. Shape-changing legs, as opposed to shape-changing bodies, have also been explored. A sprawl-tuned autonomous robot (STAR) uses variable leg sprawl angle to adapt its stiffness and height^50^. Passively telescoping legs on another robot can compress in a programmed direction and allow it to passively traverse narrow channels^51^. Other examples of folding robots include a compliant legged articulated robotic insect (CLARI)^18^, a cockroach-inspired soft legged robot^52^, a hybrid mobile robot with controllable stiffness^53^, and a foldable drone^54^. However, these works do not explore the potential for mobile manipulation of obstacles.

The ability to change shape allows robots to expand their functionality and application due to enhanced agility and adaptability. Enabling shape sensing, automating shape-changing, and integrating functional materials into systems remain overarching challenges in their development^55^. Our transformable structure uses rigid-body kinematics which can actively change shape. Other advantages of the design include the potential to carry payload and additional sensor modalities. We believe the robot offers advancement through the proposed combination of predictive (vision), reactive (proprioception), and active (manipulation) obstacle traversal strategies.

## Method

**Fig. 2:**
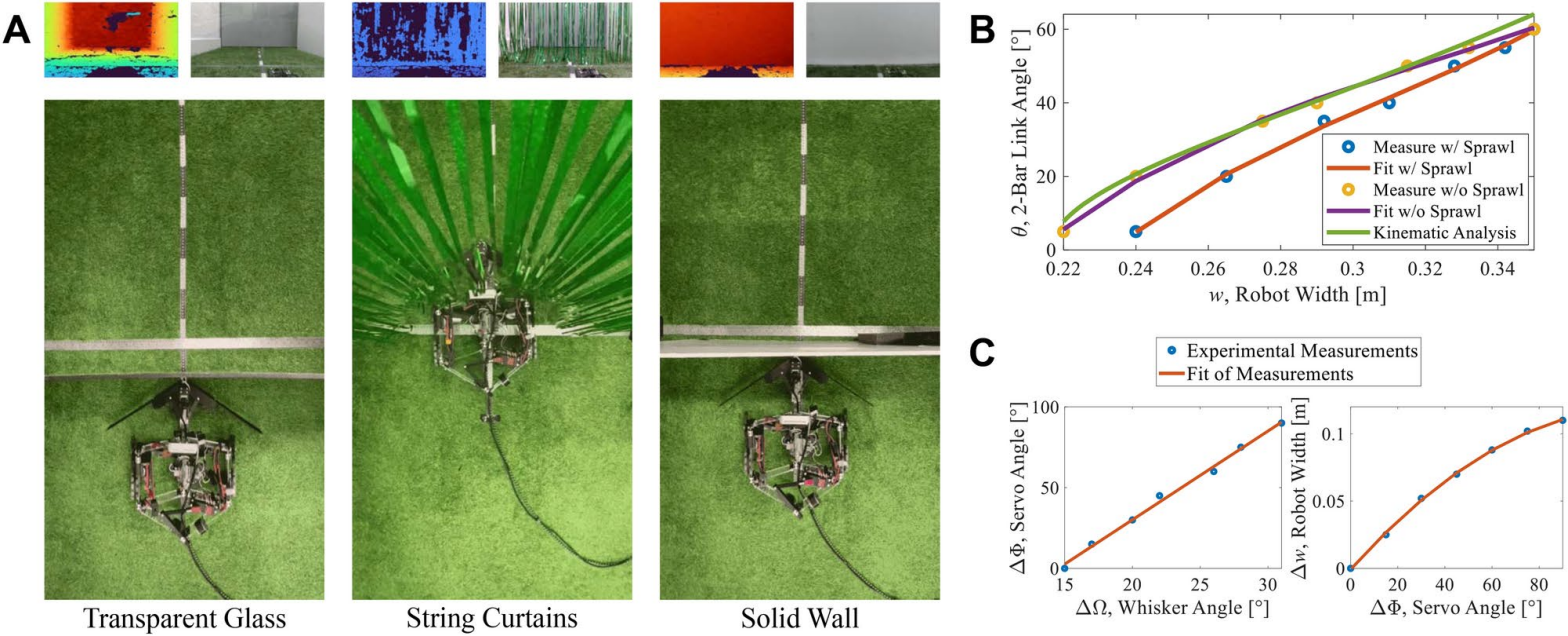
(**A**) Edge cases illustrating the motivation for employing multimodal sensor fusion. The top row shows the relative colourmap depth and RGB images from the robot’s onboard camera, while the bottom row shows the robot’s progression during the navigation attempt. (**B**) Validation of the kinematic analysis. The cubic expressions for experimental measurements of ${\theta }$ (the 2-bar link angle) given *w* (the width of the robot) are plotted against the kinematic equations derived. (**C**) Relationships between the change in whisker angle ${\Delta \Phi }$ and change in servo angle ${\Delta \Phi }$ are modelled by linear regression (left), and between the change in servo angle ${\Delta \Phi }$ and change in robot width ${\Delta }w$ are modelled by quadratic polynomial regression (right).

To highlight the motivation for using haptic and visual sensing modalities, we tested the robot’s reaction to various edge cases where single sensor modality fails. The visual prediction and navigation attempts of obstacles such as transparent glass, string curtains, and a solid wall are shown in Fig. 2A. The robot does not recognize the transparent glass as an obstacle, whereas it identifies both the string curtains and the solid wall as being occupied. In tests, recorded angle measurements show there are no whisker angle changes, highlighting that these are complicated situations which need more than one modality. While vision is desired for allowing the robot to prepare its body shape, speed, and strategy for navigation, proprioceptive sensing is needed to confirm that the robot can physically traverse obstacles in different circumstances without causing damage to itself or its environment and without reducing efficiency by attempting to accomplish impossible tasks. Manipulation can be used if the robot gets stuck or when the biggest gap in the environment is smaller than the robot’s narrowest possible width.

We utilise an enhanced version of DeforMoBot, a bio-inspired deformable mobile robot^24^. A number of augmentations have been made to the robot to expand its capabilities, as detailed in Fig. 1B; an Intel RealSense Depth Camera provides depth and RGB sensing, while a microswitch at the front tip of the robot improves proprioceptive sensing. Speed and direction control are also added for different approach strategies. The whiskers are elongated for multiple reasons: the wheels and robot body are shielded during obstacle interaction, obstacles are better protected during navigation (instead of being in contact primarily with the rimless wheels), and the robot uses the whiskers to ensure that it can push directly against objects when it expands to manipulate obstacles and gap sizes. The shape of the entire robot is controlled solely by the robot’s 2-bar link angle ${\theta }$. This value can be calculated in terms of the desired robot width *w* as shown in the Supplementary Methods.

Figure 2B compares the predicted 2-bar link angle ${\theta }$ based on the desired robot width *w* from the kinematic equations and the experimental measurements of ${\theta }$ for different *w* values with and without sprawl. As expected, the influence of sprawl increases as the robot width decreases. The overlap of the data plotted in the figure demonstrates a good fit between experimental data and the analytical predictions.

The relationships between the robot angles and width are shown in Fig. 2C. The left-hand plot shows the independent tuned linear relationship between the change in the whisker angle ${\Delta \Omega }$ and the change in the servo angle ${\Delta \Phi }$ which solely controls the robot’s shape. This tuning must complement the robot’s body shape to achieve meaningful behaviour: whisker perception that is too sensitive would result in overcompensation by the robot compressing its body shape to traverse obstacles, while whisker perception that is overly coarse would leave the robot at risk of struggling to move through gaps. The direct quadratic relationship between the servo angle ${\Delta \Phi }$ and the change in the robot’s width ${\Delta }$w is plotted on the right-hand side of Fig. 2C.

### Haptic And Visual Environment Navigation (HAVEN) Architecture

**Fig. 3:**
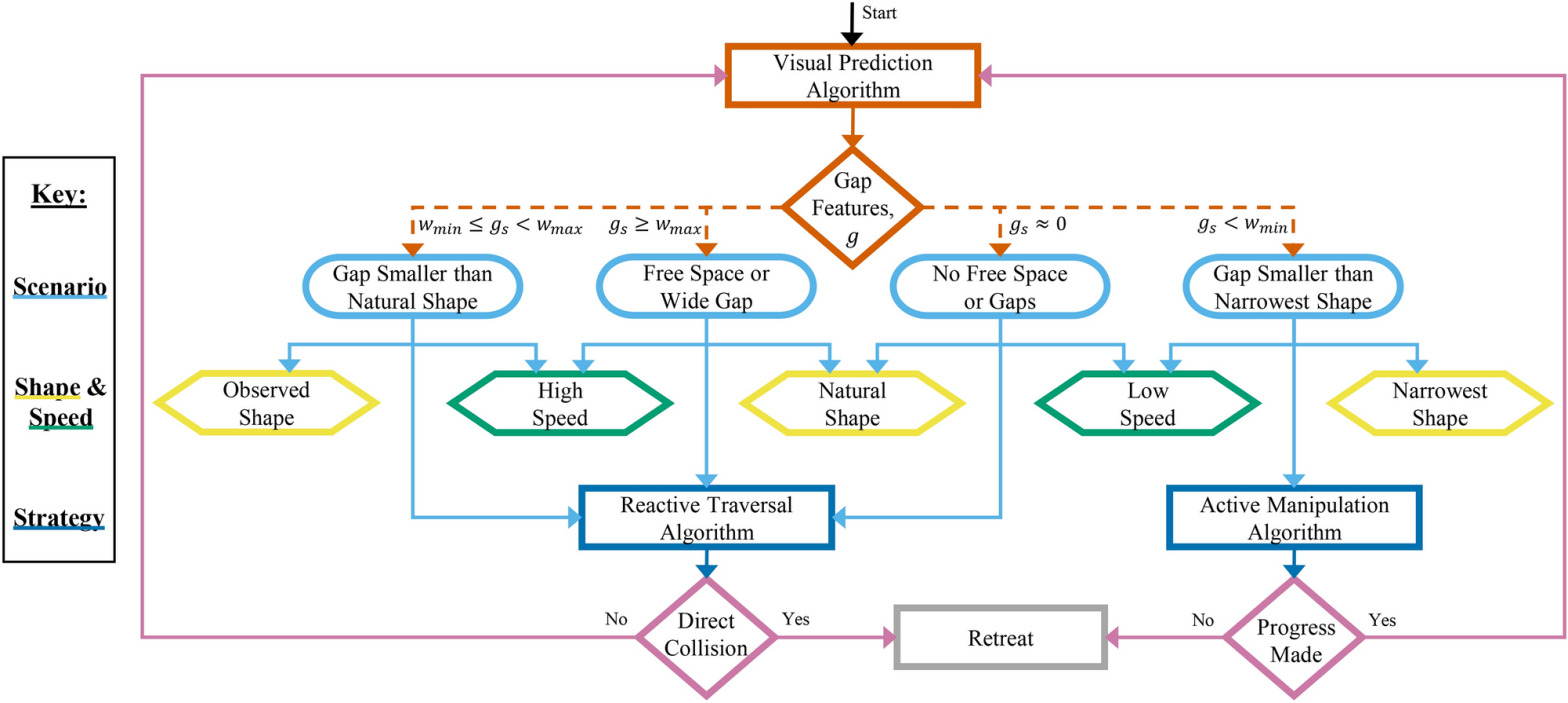
Haptic and visual environment navigation (HAVEN) architecture. After determining a state (light blue ellipses) based on the output of the Visual Prediction Algorithm (in orange), the robot autonomously chooses its initial shape (yellow hexagons), driving speed (green hexagons) and navigation strategy (dark blue rectangles). Diamonds represent decision points, ellipses show states, hexagons depict processes, and rectangles portray algorithms.

We present the HAVEN Architecture in Fig. 3 which the robot employs to autonomously make decisions and take actions. The Visual Prediction Algorithm enables the robot to be *predictive* by analysing the environment and preparing for the estimated conditions before it encounters them. The Reactive Traversal Algorithm allows the robot to be *reactive* to real-time proprioceptive feedback in the course of its journey. The Active Manipulation Algorithm enables the robot to *actively* control its environment by manipulating obstacles and gap sizes.

The Visual Prediction Algorithm uses the depth camera and operates as follows. A ROS node subscribes to depth data messages in real-time and converts these to OpenCV image encoding, focusing on a region of interest. The algorithm then smoothens out “depth holes” in the images using mean value replacement and determines the existence of gaps between obstacles by grouping together similar depth readings, *d*, within a threshold, $d_{th}$. If any apertures exist in the depth image, various parameters are calculated such as the start and end pixel indices so that the biggest gap can be calculated.

The biggest gap distance is converted from pixels to meters by first extracting the intrinsic calibration matrix, *K*, containing the focal length $(f_{x},f_{y})$ and optical centre $(c_{x},c_{y})$. The average point clouds at the start and end of the aperture are calculated by converting (*x*, *y*) pixel coordinates to 3D point coordinates in the camera frame.

1 $$\begin{aligned} point = \begin{bmatrix} d(x-c_{x})/f_{x} \\ d(y-c_{y})/f_{y} \\ d \\ \end{bmatrix} \end{aligned}$$

This imparts the depths of the obstacles on either side and ensures that the algorithm can be applied to scenes regardless of the obstacle positions or approach angle of the robot.

**Fig. 4:**
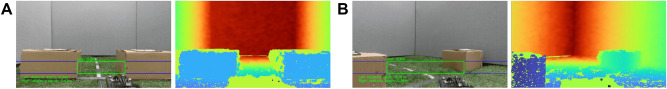
Examples of the Visual Prediction Algorithm employed by the robot from different approach angles on various obstacles with different apertures: (**A**) a $20~\textrm{cm}$ gap between weighted boxes approached directly from ${90}^{\circ }$, and (**B**) a $40~\textrm{cm}$ gap between weighted boxes approached from an angle of ${45}^{\circ }$. In each group, the coloured depth images are shown on the left and the aligned depth-to-colour images with overlaid feature information are shown on the right.

The Euclidean distance, $g_{s}$, between the two 3D points is calculated and communicated to the robot so that it can decide how best to proceed. The initial shape of the robot is set as

2 $$\begin{aligned} \hat{\Phi }_{t=0} = \left\{ \begin{array}{ c l } \Phi _{min}, & \quad \text {if } g_{s} \ge w_{max} \\ \Phi _{max}, & \quad \text {if } g_{s} \le w_{min} \\ a g_{s}^{2} + b g_{s} + c, & \quad \text {{otherwise}} \end{array} \right. \end{aligned}$$

where *a*, *b*, and *c* are coefficients which can be adjusted depending on application. In our case, we use the method of least squares to obtain coefficients of $a = 3925$, $b = -1540$, and $c = 200$ which satisfy the desired relationship. Fig. 4 shows sample depth and aligned depth-to-colour images overlaid with gap bounding boxes, gap widths, and obstacle depths.

There are four possible outcomes to the Visual Prediction algorithm outlined as follows.

1. In free space, or where the gap is bigger than the robot’s natural shape, the robot progresses in its natural shape and follows an Obstacle Traversal Algorithm inspired by the shape/mobility algorithm previously introduced^24^. It updates its shape using proprioceptive feedback and retreats if it detects head-on collision with an unseen obstacle. This can occur in edge cases such as windows or transparent glass.
2. If no free space or gaps are identified, the robot proceeds cautiously at low speed, again employing the Obstacle Traversal Algorithm and retreating if a head-on collision occurs. This addresses cases such as obstacles which may be solid (e.g. walls) or deformable (e.g. string curtains), and also in the event of camera failure.
3. If the largest aperture measures between the robot’s smallest and largest widths, the robot changes size accordingly to match this observed distance and executes the Obstacle Traversal Algorithm once again, retreating in the case of a head-on collision. It updates using proprioceptive feedback and resumes its natural shape once it has traversed the obstacles.
4. Finally, if the biggest opening is narrower than the robot’s smallest size, it drives slowly at its narrowest width and follows the Obstacle Manipulation Algorithm presented in the supplementary material. After the robot comes into contact with the obstacles (determined by proprioceptive whisker feedback), it changes the servo angle by a degree at a rate of $200~{\textrm{ms}}$ to gradually widen its body shape to push against the obstacles and expand the gap. If the robot can widen the gap enough to fit through, it can traverse the obstacles before resuming its natural shape. If it cannot manipulate the gap and progress within a certain time frame, it retreats and can look for other possibilities if desired.

## Experiments and results

Several experiments were performed to analyse the robot’s performance following the HAVEN Architecture. These experiments compared the traversal and manipulation strategies in different scenarios and also tested the robot in different obstructed, challenging environments.

Having no prior knowledge of the scene, obstacles, or aperture size, the robot used the Visual Prediction Algorithm. Examples of depth and aligned depth-to-colour images obtained for different obstacles are shown in Fig. 4. Colourmaps are applied to the depth images to clearly show the relative depth measurements, and details of feature information are overlaid on the RGB images including the obstacle depths measured, the largest gaps seen in the images, and the “bounding boxes” of these gaps. For reference, depth and aligned depth-to-colour images with overlaid features for all obstacle types at each approach angle are shown in Supplementary Fig. 1.

### Comparison of Traversal and Manipulation Algorithms

The Traversal and Manipulation Algorithms were tested and compared in different scenarios. We used balls, boxes containing 2.5 kg, cushions, and stone blocks as obstacles since these cover a wide range of physical properties. The robot was tested attempting to traverse gaps between the obstacles of $40~{\textrm{cm}}$, $30~{\textrm{cm}}$, $20~{\textrm{cm}}$, and $10~{\textrm{cm}}$ (ranging from bigger than its natural size of $w_{max}=35~{\textrm{cm}}$ to much smaller than its narrowest width of $w_{min}=24~{\textrm{cm}}$) from angles of ${90}^\circ$ (directly facing them) and ${45}^{\circ }$.

We also conducted additional tests involving the robot blindly driving full speed fixed in its Natural shape and in its Narrowest shape. In its Natural shape, the robot achieves comparatively lower success rates and higher navigation times. In its Narrowest shape, it performs similarly to the Traversal strategy, but without the aforementioned benefits of shape change. There is also likely to be attrition on the robot and any payload from repeated higher-speed collisions, and there is higher likelihood that damage can be caused to the robot or its environment. The navigation time statistics are shown in Supplementary Fig. 2 with direct comparison of the median navigation times obtained in Supplementary Table 1. Images of the robot’s progression for each of these cases—at all gap sizes for every obstacle at each approach angle—are included in Supplementary Figs. 3–6.

In order to directly compare the reactive Obstacle Traversal Algorithm and the active Obstacle Manipulation Algorithm, we constrained the robot to employ each algorithm in every scenario where apertures are smaller than the robot’s natural width. The robot still autonomously controlled its initial conditions based on the visual prediction. As it progressed, it continued to adjust its motion and shape based on real-time proprioceptive feedback from the whisker and microswitch measurements, and was allowed up to 15 seconds for navigation.

**Fig. 5:**
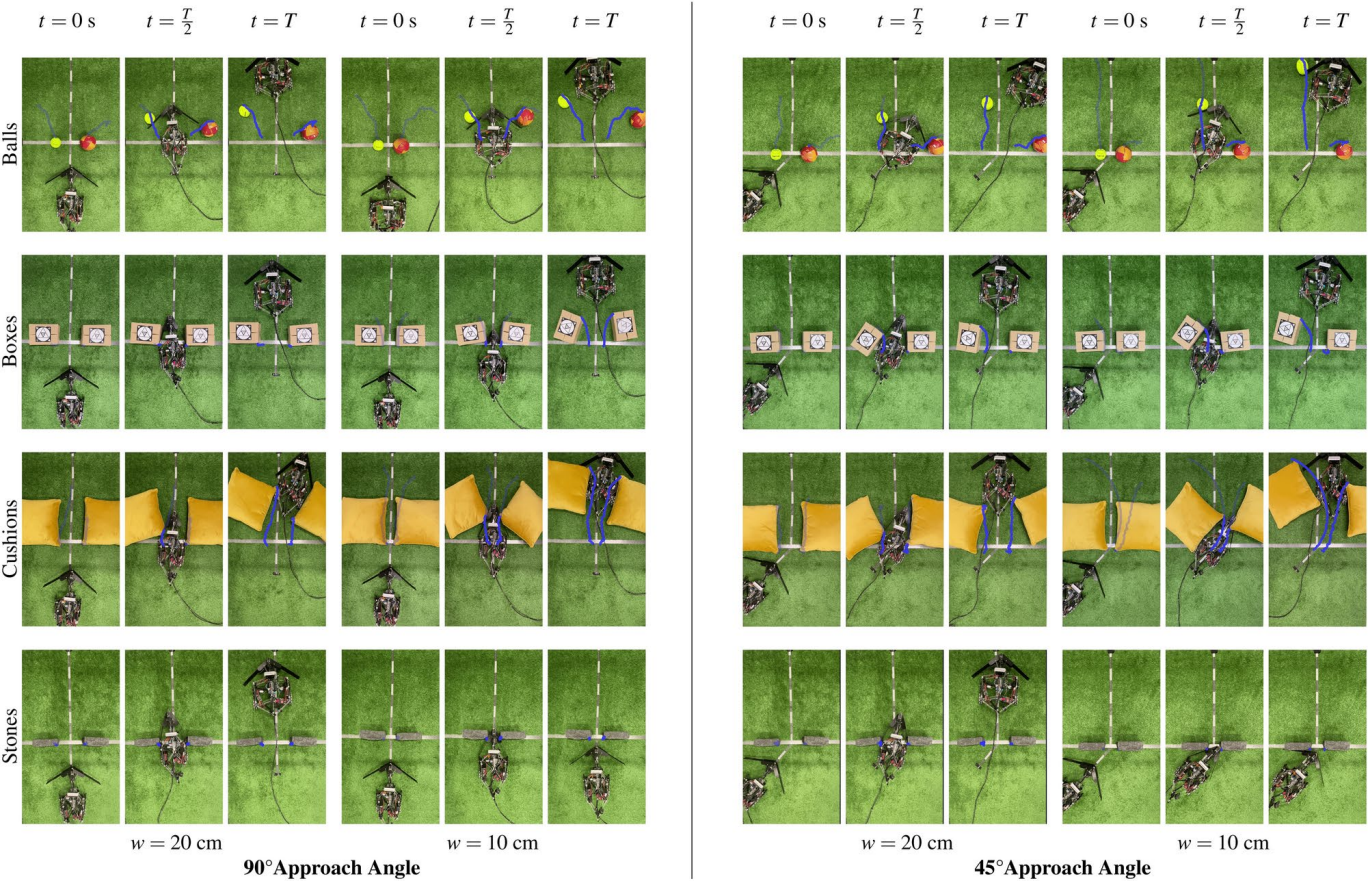
The robot attempts to traverse various obstacles employing the Obstacle Manipulation Algorithm in which it uses its shape-changing ability to push against the obstacles. The obstacles’ movements are annotated in blue to clearly show the effects of the robot’s interaction with them. Timestamps are shown in the column headers, obstacles are listed in the row headers, and gap widths are detailed below the figures.

The robot was afforded 10 attempts at traversing each obstacle, at each width, from each approach angle, using each algorithm, resulting in a total of 480 trials overall. The progression of the robot’s path and shape as it employs the Obstacle Manipulation Algorithm at gap sizes smaller than its narrowest possible width is shown in Fig. 5. The obstacles’ movements are annotated in blue to clearly show the effects of the robot’s interaction with them, and the average total displacement of these obstacle points are shown in Supplementary Table 2. For comparison with the Obstacle Manipulation Algorithm, we include images of the robot’s progression as it exclusively used the Obstacle Traversal Algorithm—at all gap sizes for every obstacle at each approach angle—in Supplementary Figs. 7 and 8.

**Fig. 6:**
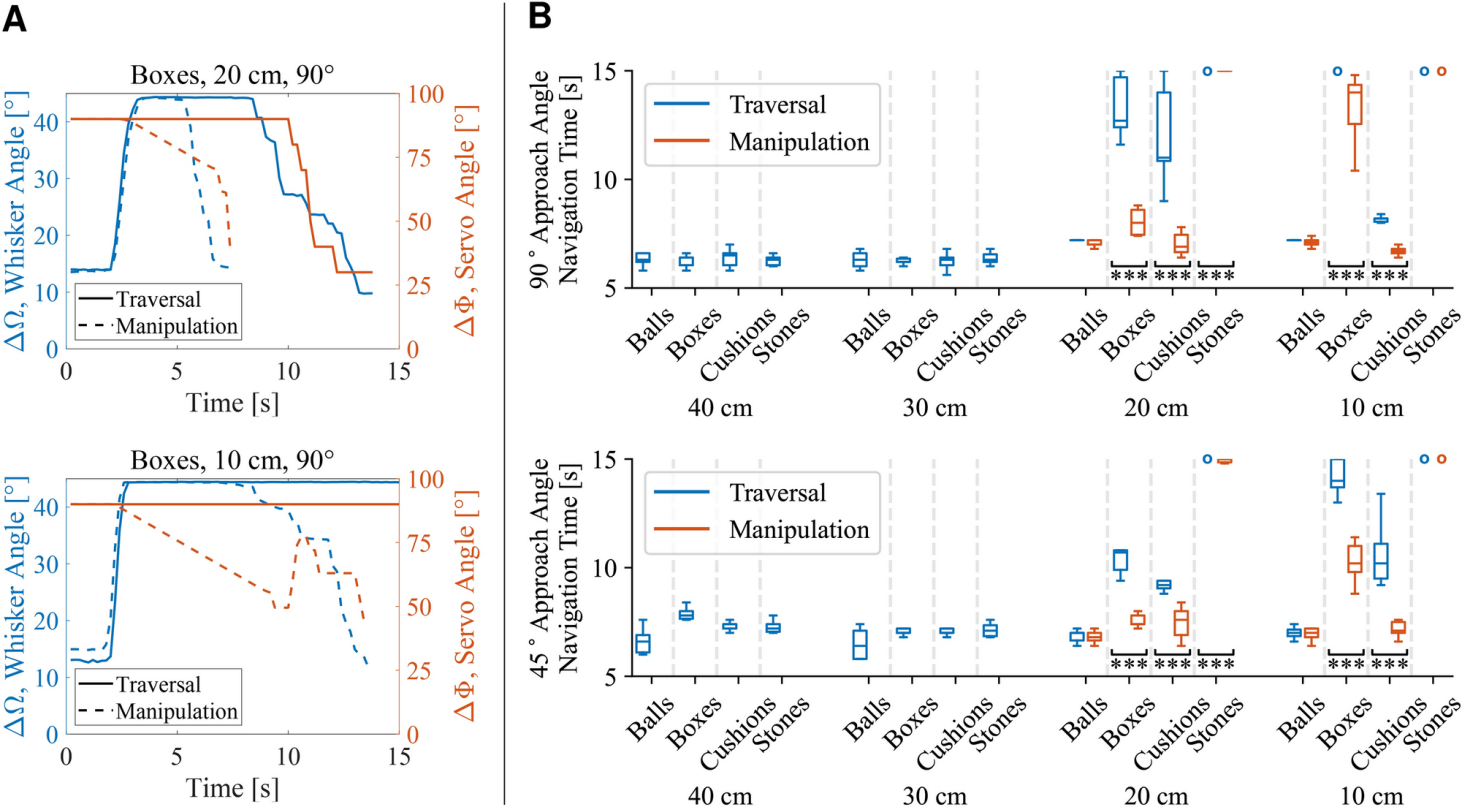
(**A**) Direct comparisons of the mean measurements of the whisker angle $${\Delta \Omega }$$ (in blue) and servo angle $${\Delta \Phi }$$ (in orange) of the robot employing the Traversal and Manipulation Algorithms (detailed in the legend) for a particular obstacle (in this case, weighted boxes) at apertures of $$20~{\textrm{cm}}$$ (top plot) and $$10~{\textrm{cm}}$$ (bottom plot) approached directly at $${90}^{\circ }$$. The robot’s initial body shape (controlled by the servo angle $${\Phi }$$) is set using visual prediction and updates based on proprioceptive feedback. (**B**) Box plots comparing summary statistics of experiments involving the robot employing the Traversal (in blue) and Manipulation Algorithms (in orange) for the various scenarios. Each box extends from the lower to upper quartile values of the data, with a line at the median. Whiskers extend from boxes to show the range of the data. Circles represent failed navigation attempts. Plots show results of paired two-tailed Mann–Whitney U tests where $$^{*}p<0.05$$, $$^{**}p<0.01$$, and $$^{***}p<0.001$$.

Examples of the robot’s whisker angle changes ${\Delta \Omega }$ and servo angle changes ${\Delta \Phi }$ for a particular case are presented in Fig. 6A, with other obstacles, apertures, and approach angles shown for reference in Supplementary Fig. 9. The servo angle—controlling the robot’s overall shape—is reactive in real-time to feedback from the whisker apart from a rewidening delay so that the robot does not become stuck when progressing through obstacles. It begins at an angle determined by the results of the Visual Prediction Algorithm and holds this position until it comes into contact with the obstacles when it can then update in real-time.

**Table 1:**
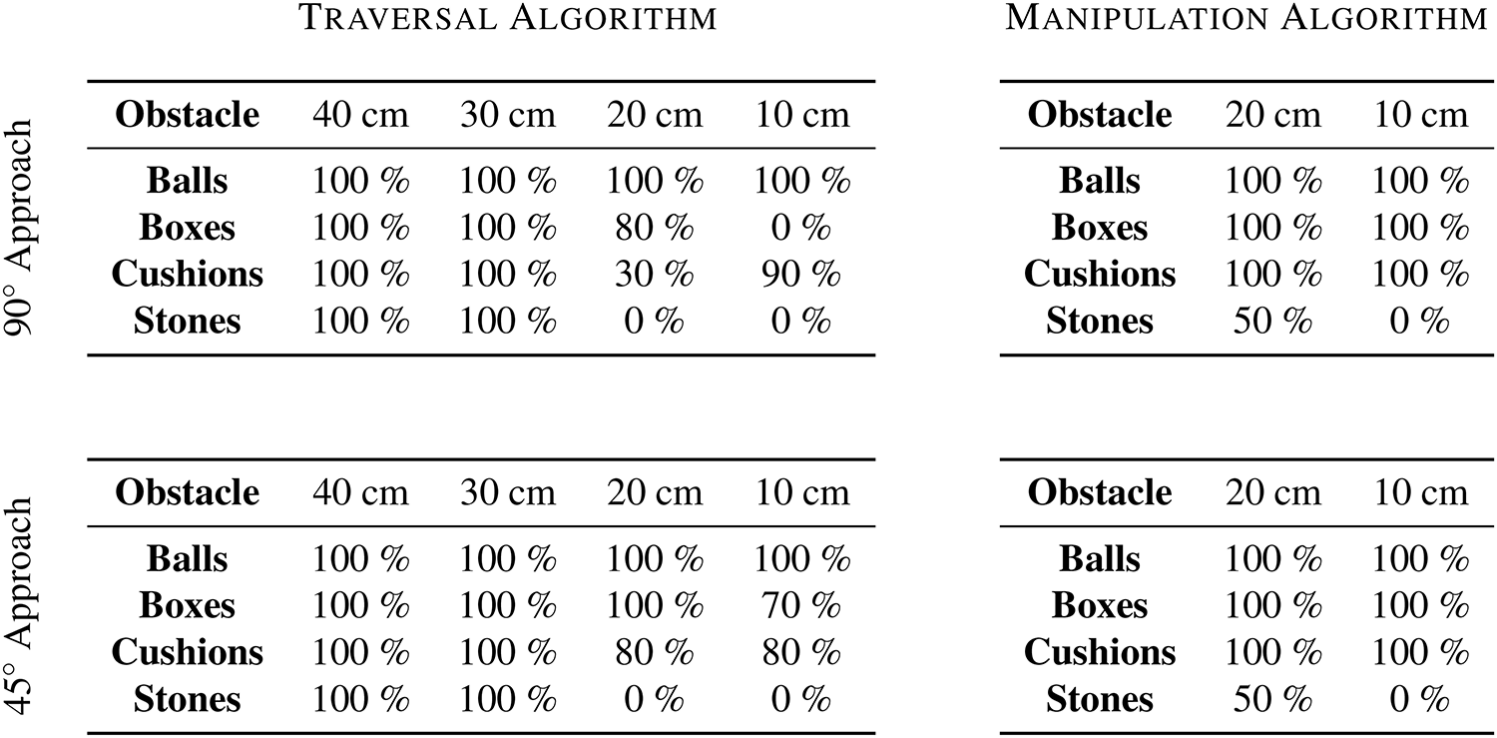
Navigation success rates.

The success rates from the experiments are summarised in Table 1 and the navigation time statistics are shown in Fig. 6B. The robot had excellent navigation results of 100% success for all obstacles at wider apertures of $w=40$ and $30~{\textrm{cm}}$. It achieved 100% success at negotiating balls at all gap sizes since they rolled away upon contact and caused insignificant whisker deformation. Narrower gap distances between other obstacles proved more challenging as expected.

The Manipulation Algorithm outperformed the Traversal Algorithm for apertures narrower than the robot’s smallest possible width. It achieved 100% success—and improved times taken—at overcoming the boxes and the cushions at every aperture, and was sometimes able to manipulate and traverse the heavier stone blocks. Given longer or multiple obstacle manipulation attempts, the robot could potentially achieve even higher navigation success rates.

**Table 2:**
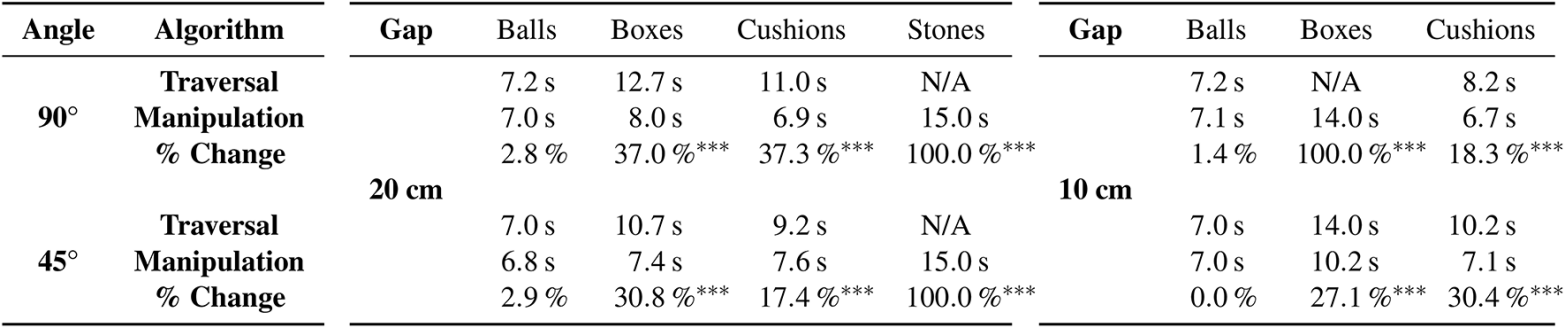
Comparison of median navigation times.

Results of paired two-tailed Mann–Whitney U tests where $$^{*}p<0.05$$, $$^{**}p<0.01$$, and $$^{***}p<0.001$$

Table 2 further examines the results obtained by comparing the median navigation times of the algorithms and performing paired two-tailed Mann–Whitney U tests. The Obstacle Manipulation Algorithm achieved lower navigation times in all cases involving the narrower gaps from both approach angles of 90°and 45°: for gaps of $20~{\textrm{cm}}$, it achieved improvements for boxes of 37.0% ($p<0.001$, Mann–Whitney U test) and 30.8% ($p<0.001$), for cushions of 37.3% ($p<0.001$) and 17.4% ($p<0.001$), and for stones of 100% ($p<0.001$) in both cases, respectively; for gaps of $10~{\textrm{cm}}$, it accomplished improvements for cushions of 18.3% ($p<0.001$) and 30.4% ($p<0.001$), and for boxes of 100% ($p<0.001$) and 27.1% ($p<0.001$), respectively.

### Outdoor environments

**Fig. 7:**
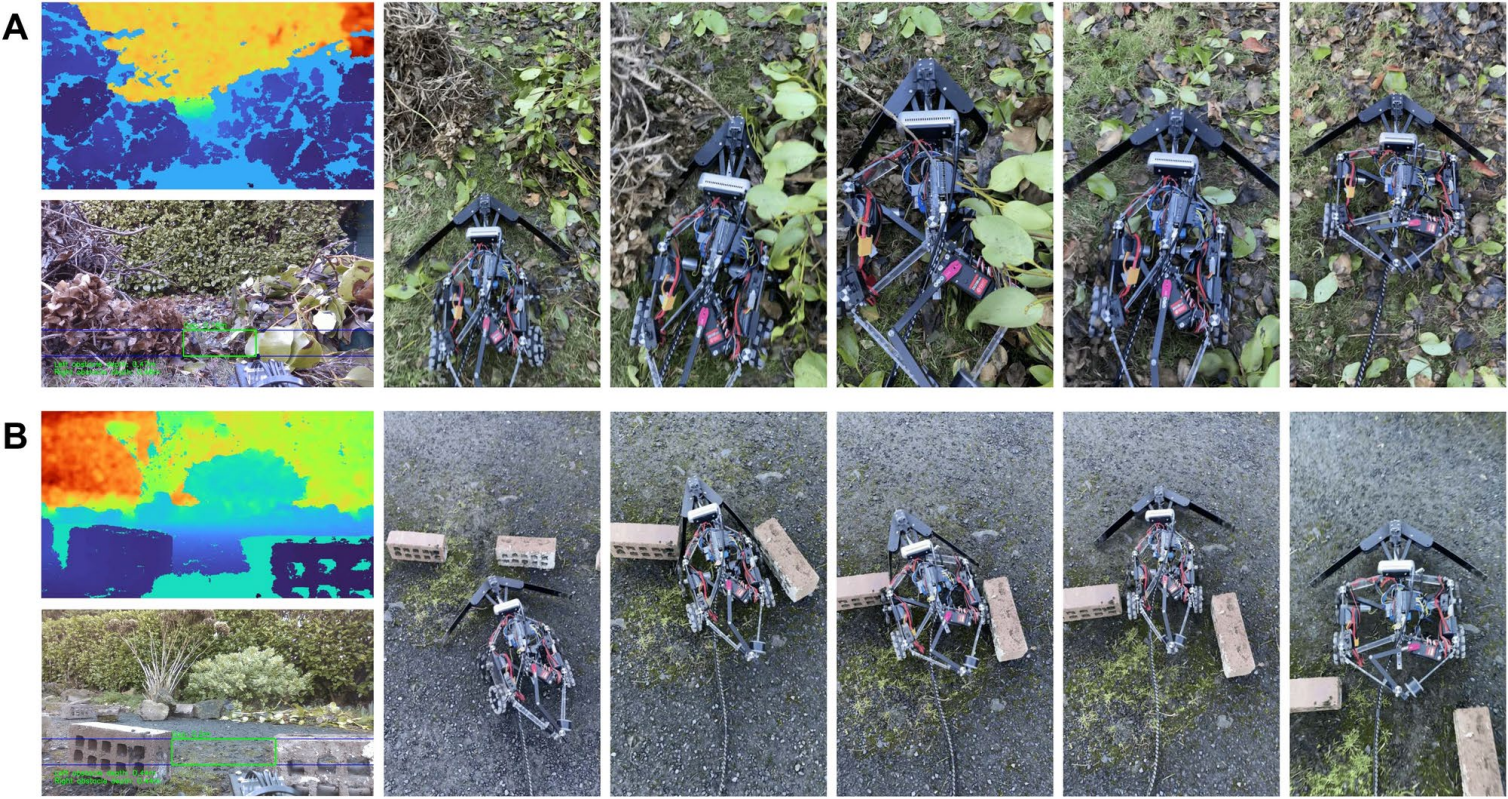
Examples of the robot using the HAVEN Architecture to progress through gaps narrower than its minimum size between obstacles in outdoor environments such as twigs and leaves (**A**), and bricks (**B**). From left-right: the robot analyses the coloured depth images (top) and aligned depth-to-colour images with overlaid information (bottom), approaches at its narrowest width, and since it cannot fit through, it expands its body shape to widen the gap, and is then able to compress once more and progress, before finally resuming its natural shape.

Finally, the robot and its algorithms were observed in different outdoor environments including terrains such as tarmac, gravel, and grass, and with obstacles including leaves, stones, rocks, and bricks. Examples of such tests are shown in Fig. 7 in which the robot attempts to traverse gaps between obstacles comprising twigs and leaves (Fig. 7A) and bricks (Fig. 7B). Following the HAVEN Architecture, the robot observed the gap was smaller than its minimum width and chose its navigation strategy accordingly. By pushing the obstacles apart using its shape-changing body, it was able to create a gap wide enough for it to progress through before resuming its natural shape.

The capabilities of the robot and algorithms presented result in improved perception and proficiency, allowing the robot to navigate more effectively and efficiently. These abilities can be particularly useful in obstructed, cluttered, unstructured, or otherwise challenging environments. The ability of the robot to manipulate obstacles could also be useful for widening gaps to allow other bigger robots to traverse. Furthermore, twins or multiples of the robot could potentially act together and unite to manipulate larger or heavier obstacles. The aforementioned ideas, further perception and sensing modalities, and marginal design improvements will be interesting to explore in future work.

## Conclusions

This paper presents a new Haptic And Visual Environment Navigation (HAVEN) Architecture for predictive, reactive, and active obstacle traversal and manipulation strategies by a deformable mobile robot using vision and proprioceptive sensing. We tested the robot attempting to traverse obstructed environments with different types of obstacles placed apart at decreasing gaps from $w=40~{\textrm{cm}}$ to $w=10~{\textrm{cm}}$. These obstacles varied in their geometric and mechanical properties, and gaps between them were on a scale ranging from greater than the robot’s natural widest shape of $35~{\textrm{cm}}$ to much smaller than the robot’s narrowest width of $24~{\textrm{cm}}$. The proposed approach of using visual feature extraction to choose between reactive obstacle traversal and active obstacle manipulation results in achieving high success rates (detailed in Table 1) and a significant reduction in time (shown in Table 2) to negotiate gaps. This improves the robot’s locomotion and navigation abilities, particularly in obstructed environments.

## Supplementary Information

Supplementary Information 1.

Supplementary Information 2.

Supplementary Information 3.

## Data Availability

The data supporting the findings of this study are in the main text and supplementary materials. Raw data and code are available from the corresponding author upon reasonable request.
