# Supplementary material for: HAVEN: Haptic And Visual Environment Navigation by a Shape-Changing Mobile Robot with Multimodal Perception
Source: Sci Rep. 2024 Nov 6;14:27018. doi: 10.1038/s41598-024-75607-7 (PMC11541753; doi:10.1038/s41598-024-75607-7)
Supplement: Supplementary file 1 — Supplementary Information 1. [file 41598_2024_75607_MOESM1_ESM.pdf]

Supplementary Material

Supplementary Video 1 (Summary): https://youtu.be/gvs9hy38KLM
Supplementary Video 2 (Experiments): https://youtu.be/JN-QqbUxQJY

Supplementary Methods

The width of the robot, as shown in Fig. 1B, is

$$w = 2w_{wl} + 2l_1 \sin\frac{\Theta}{2}, \tag{1}$$

where w_{wl} is the width of each wheel and Θ is the angle of deformation of the robot body. The robot angle Θ can also be derived, using the law of sines, as

$$\Theta = 2\sin^{-1}\left(\frac{l_2 \sin(\alpha+\beta)}{l_k}\right), \tag{2}$$

where, by laws of geometry,

$$l_k = (l_1 + l_2)\sin\frac{\alpha+\beta}{2}, \tag{3}$$

$$\alpha = \cos^{-1}\frac{l_1^2 + l_9^2 - l_8^2}{2l_1 l_9}, \tag{4}$$

and

$$l_8 = 2l_6 \cos\Theta. \tag{5}$$

Rearranging (1) and equating with (2),

$$\Theta = 2\sin^{-1}\left(\frac{w - 2w_{wl}}{2l_1}\right) = 2\sin^{-1}\left(\frac{l_2 \sin(\alpha+\beta)}{l_k}\right) \tag{6}$$

$$\Rightarrow l_k (w - 2w_{wl}) = 2l_1 l_2 \sin(\alpha+\beta). \tag{7}$$

Expanding (7) with (3), we obtain

$$\frac{l_1 + l_2}{2l_1 l_2}(w - 2w_{wl}) = 2\cos\left(\frac{\alpha+\beta}{2}\right), \tag{8}$$

and further expanding with (4) and (5) gives

$$\frac{l_1 + l_2}{2l_1 l_2}(w - 2w_{wl}) = 2\cos\left(\frac{\beta + \cos^{-1}\left(\frac{l_1^2 + l_9^2 - 4l_6^2\cos^2\theta}{2l_1 l_9}\right)}{2}\right). \tag{9}$$

Rearranging to isolate θ in terms of w results in

$$\theta = \cos^{-1}\sqrt{\frac{l_1^2 + l_9^2 - 2l_1 l_9 \cos\left(2\cos^{-1}\left(\frac{l_1+l_2}{4l_1 l_2}(w - 2w_{wl})\right) - \beta\right)}{4l_6^2}}. \tag{10}$$

Supplementary Table 1. Comparison of Median Navigation Times in Natural and Narrowest Shapes

Angle	Shape	Gap	Balls	Boxes	Cushions	Stones	Gap	Balls	Boxes	Cushions	Stones
**90°**	**Natural**	**20 cm**	6.8 s	N/A	N/A	N/A	**10 cm**	7.0 s	N/A	15.0 s	N/A
	**Narrowest**		7.2 s	13.4 s	13.0 s	N/A		6.8 s	N/A	9.0 s	N/A
**45°**	**Natural**		7.4 s	N/A	15.0 s	N/A		7.4 s	N/A	N/A	N/A
	**Narrowest**		7.2 s	11.0 s	9.2 s	N/A		7.4 s	13.6 s	7.8 s	N/A

Supplementary Table 2. Obstacle Point Average Total Displacement

	90°		45°	
**Obstacle**	20 cm	10 cm	20 cm	10 cm
**Balls**	71.91 cm	105.32 cm	116.57 cm	90.85 cm
**Boxes**	12.55 cm	68.58 cm	16.84 cm	18.92 cm
**Cushions**	91.88 cm	155.32 cm	119.31 cm	191.46 cm
**Stones**	15.88 cm	55.25 cm	13.57 cm	8.34 cm

Algorithm 1 Obstacle Manipulation Algorithm

t = current time; t_{th} = threshold time; Φ = servo angle; Ω = whisker angle; t_{pass} = elapsed time; v = speed.

```
procedure MANIPULATE OBSTACLES(t, t_th, v_max, ΔΩ_t, ΔΦ_min, ΔΦ_max, ΔΦ̂_t)
    widen, stop, delay ← False                                  ▷ Initialise flags
    v ← v_max/4                                                 ▷ Reduce speed for impact
    if (ΔΦ_max − ΔΦ̂_t) < ΔΦ_th then                             ▷ Contact made with obstacle
        if widen = False then
            t_contact ← t                                       ▷ Record contact time
        widen, stop ← True                                      ▷ Raise widening and stopping flags
        delay ← False
        v ← v_max                                               ▷ Full speed
    else
        widen ← False
------------------------------------------------------------------------
  MANIPULATE
    if widen and ΔΦ_t > (ΔΦ_min + ΔΦ_th) then
        if t_contact < t_th,1
            ΔΦ_t ← ΔΦ_t − 1                                     ▷ Interact with obstacles by widening shape
        if t_contact ≥ t_th,1
            ΔΦ_t ← ΔΦ_max                                       ▷ Compress shape to squeeze through
        if t_contact ≥ t_th,2
            retreat                                             ▷ Retreat if no progress made
------------------------------------------------------------------------
  TRAVERSE
    if widen = False then
        if ΔΦ̂_t > ΔΦ_t−1
            delay ← False
        else
            if delay = False then
                t_delay ← t                                     ▷ Record delay start time
            delay ← True                                        ▷ Raise delay flag
        t_pass ← t_delay − t                                    ▷ Calculate elapsed time since delay start time
        if delay = False or (delay and t_pass ≥ t_th,3)
            if ΔΦ̂_t < ΔΦ_min then
                ΔΦ_t ← ΔΦ_min                                   ▷ Take widest shape
            else if ΔΦ̂_t > ΔΦ_max then
                ΔΦ_t ← ΔΦ_max                                   ▷ Take narrowest shape
            else
                ΔΦ_t ← ΔΦ̂_t                                     ▷ Take desired/calculated shape
------------------------------------------------------------------------
        if stop and t_pass ≥ t_th,4 then
            v ← 0                                               ▷ Stop
end procedure
```

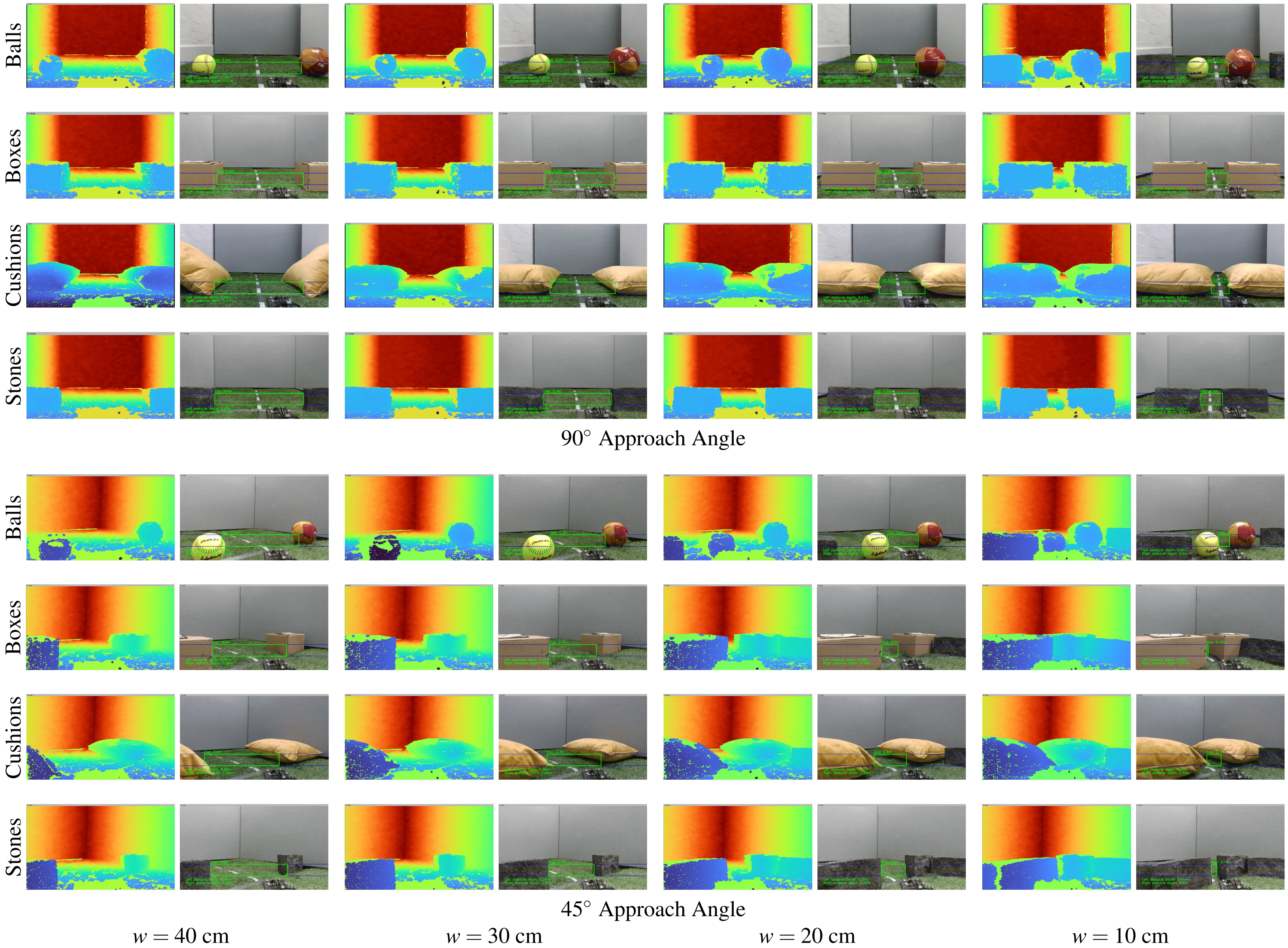

Supplementary Figure 1. Comparison of the visual gap prediction algorithm at approach angles of 90° and 45° for a variety of different obstacles with various physical properties. The obstacles are placed apart at different widths (listed below each column group) to show how the robot responds to different scenarios in relation to its own body size and navigation strategy. In each group of width columns, the depth images with depth hole filling and colormaps applied are shown on the left. On the right, the aligned depth-to-color images are shown overlaid with the depths of the obstacles, gap bounding box, and predicted gap width.

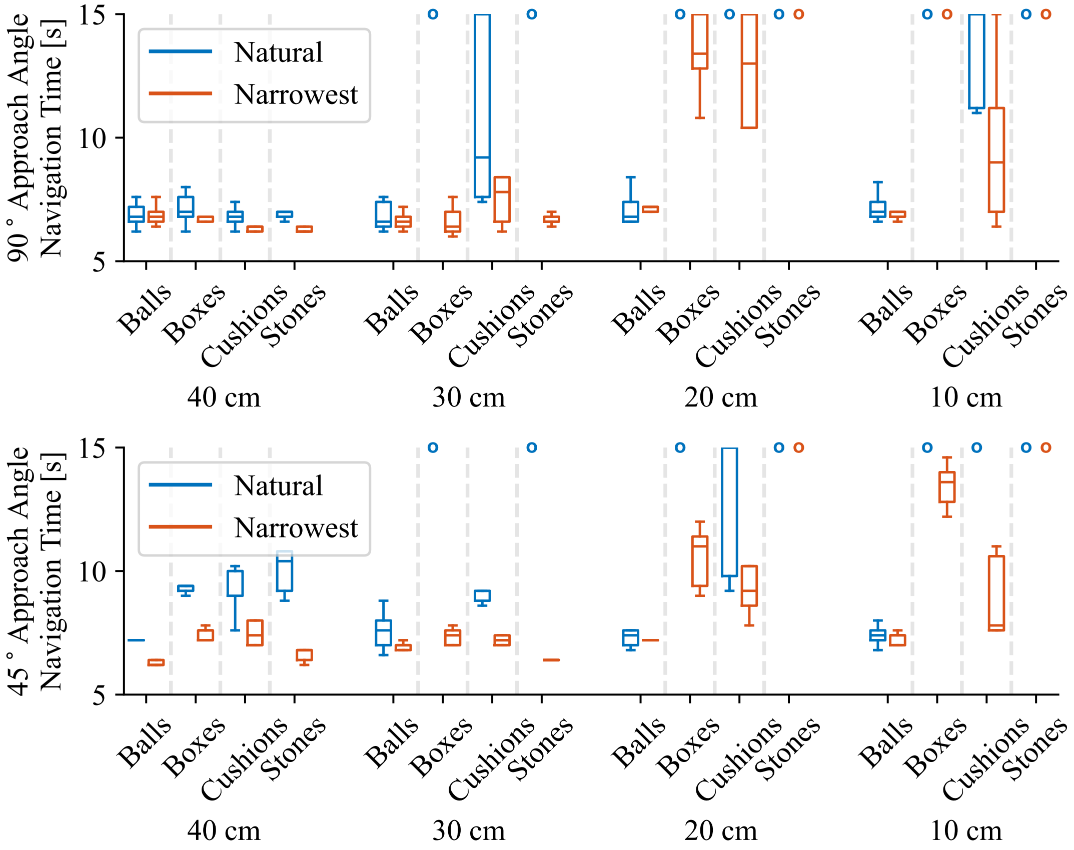

Supplementary Figure 2. Box plots comparing summary statistics of experiments involving the robot exclusively employing its Natural (in blue) and Narrowest shapes (in orange) for the various scenarios. Each box extends from the lower to upper quartile values of the data, with a line at the median. Whiskers extend from boxes to show the range of the data. Circles represent failed navigation attempts.

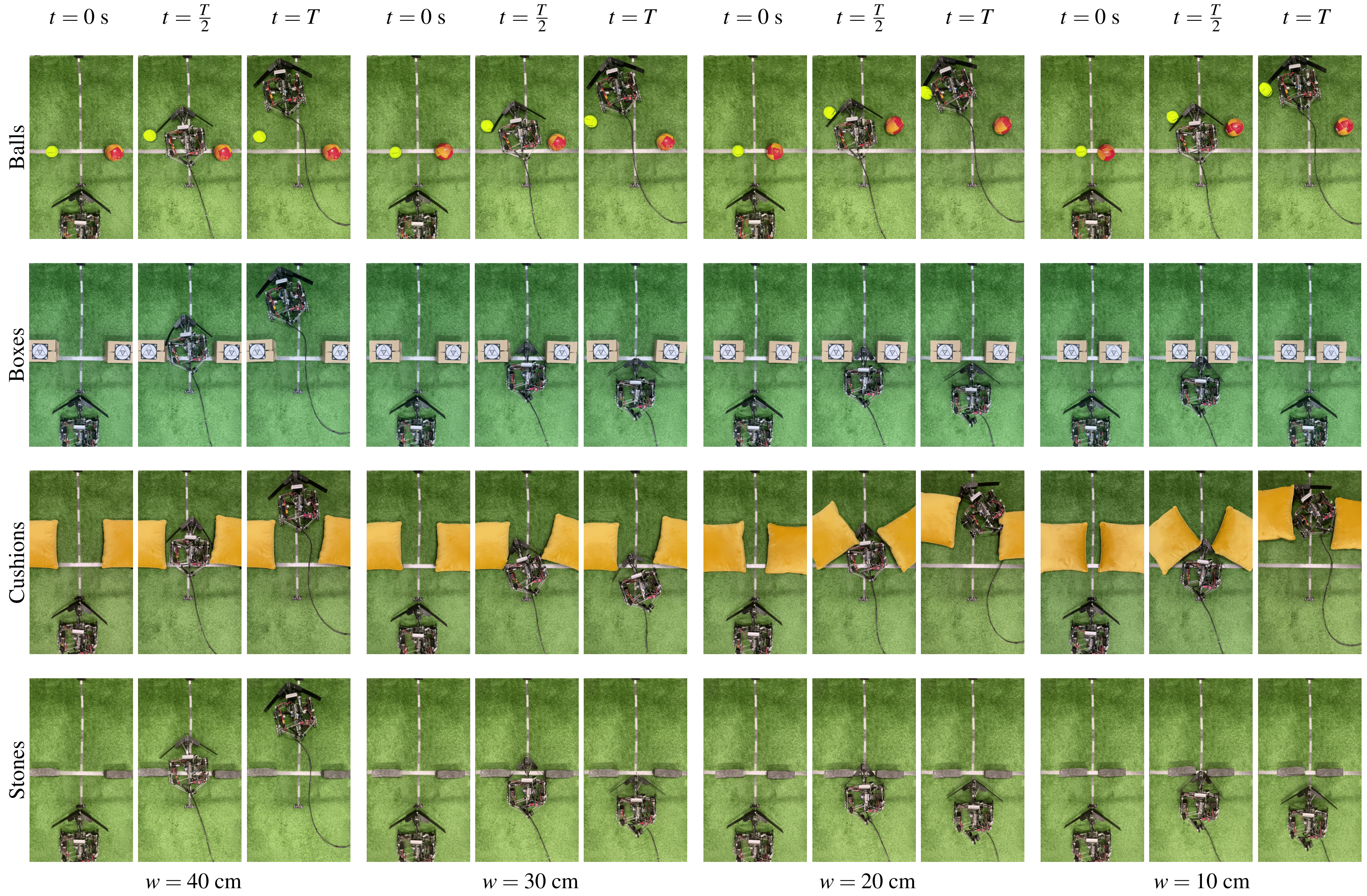

Supplementary Figure 3. Exclusively at its Natural shape, from an approach angle of 90°the robot attempts to traverse various obstacles with different physical properties placed apart by decreasing distances. Timestamps of the still frames are shown in the column headers, where T is the time it takes for the robot to complete its navigation attempt. Obstacle names are listed in the row headers and gap widths are detailed below the figures.

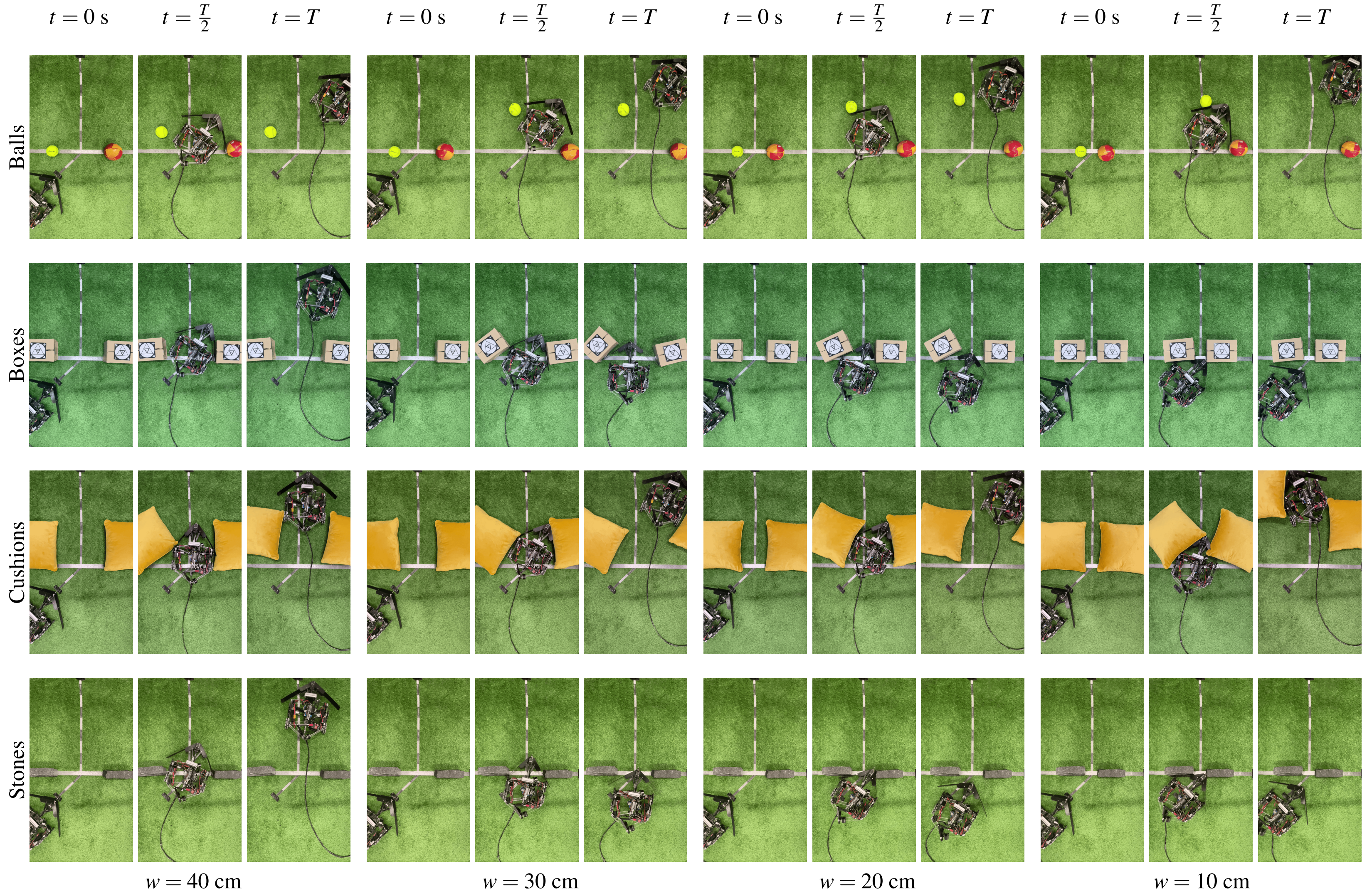

Supplementary Figure 4. Exclusively at its Natural shape, from an approach angle of 45°the robot attempts to traverse various obstacles with different physical properties placed apart by decreasing distances. Timestamps of the still frames are shown in the column headers, where T is the time it takes for the robot to complete its navigation attempt. Obstacle names are listed in the row headers and gap widths are detailed below the figures.

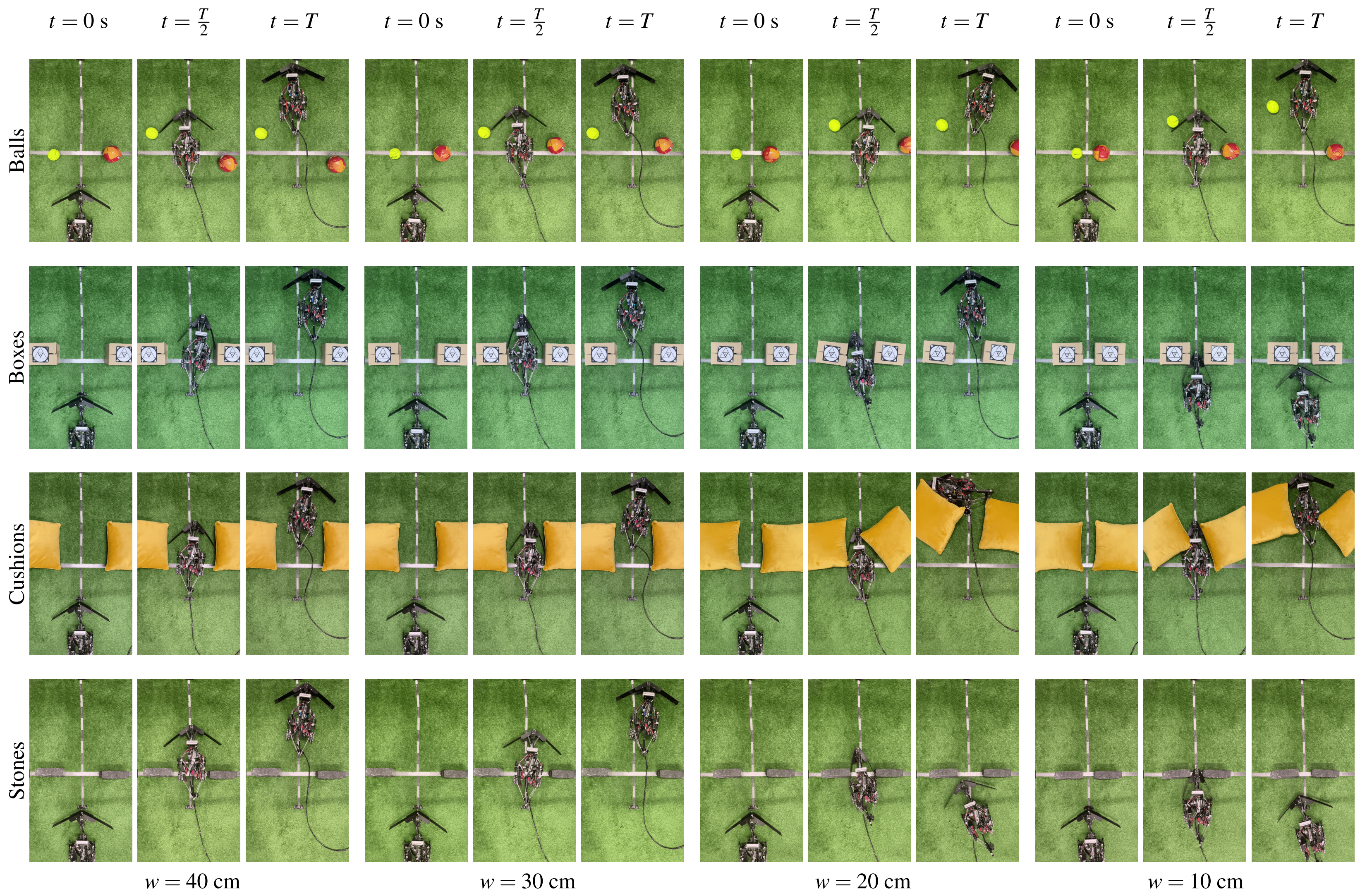

Supplementary Figure 5. Exclusively at its Narrowest shape, from an approach angle of 90°the robot attempts to traverse various obstacles with different physical properties placed apart by decreasing distances. Timestamps of the still frames are shown in the column headers, where T is the time it takes for the robot to complete its navigation attempt. Obstacle names are listed in the row headers and gap widths are detailed below the figures.

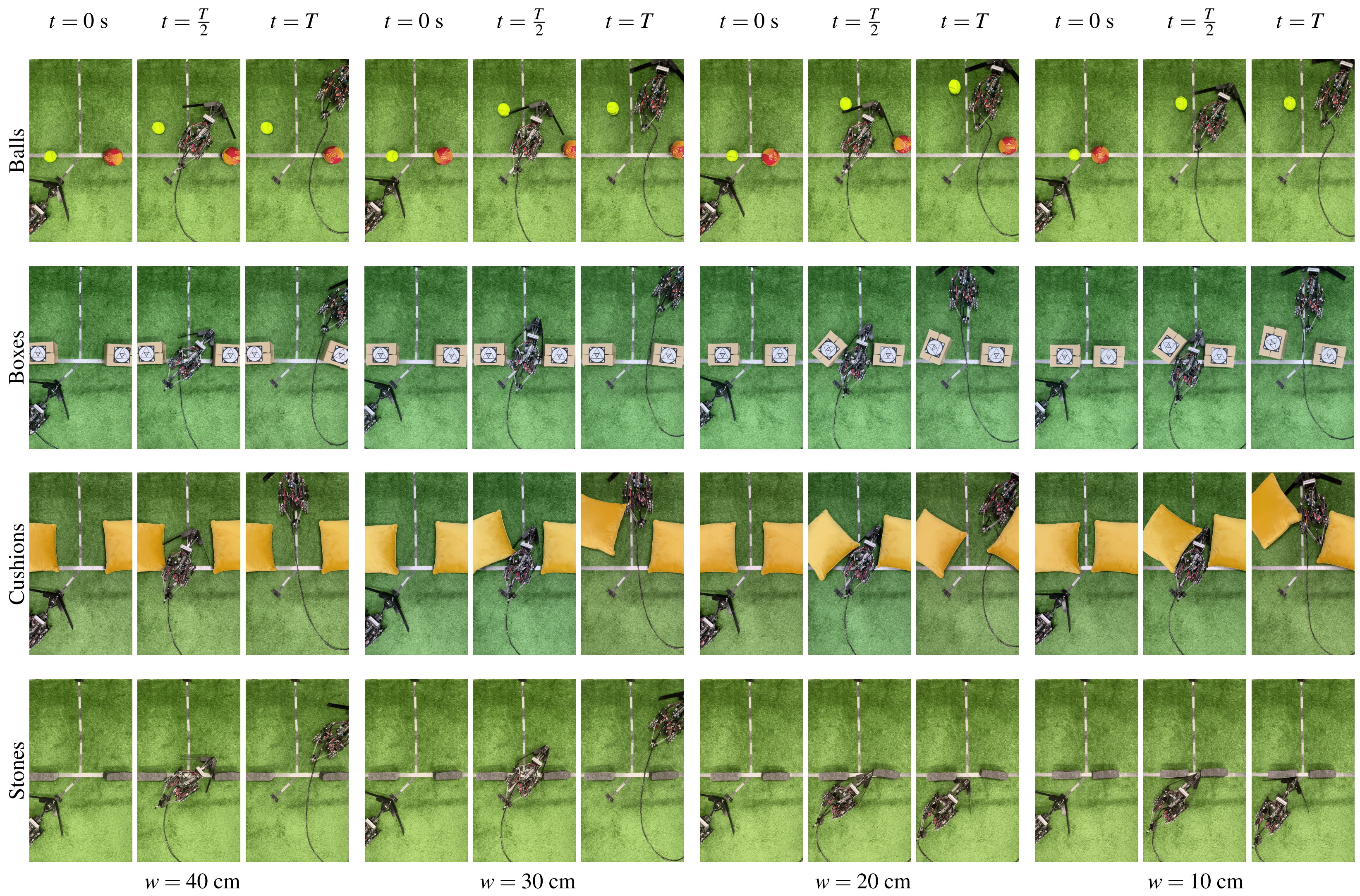

Supplementary Figure 6. Exclusively at its Narrowest shape, from an approach angle of 45°the robot attempts to traverse various obstacles with different physical properties placed apart by decreasing distances. Timestamps of the still frames are shown in the column headers, where T is the time it takes for the robot to complete its navigation attempt. Obstacle names are listed in the row headers and gap widths are detailed below the figures.

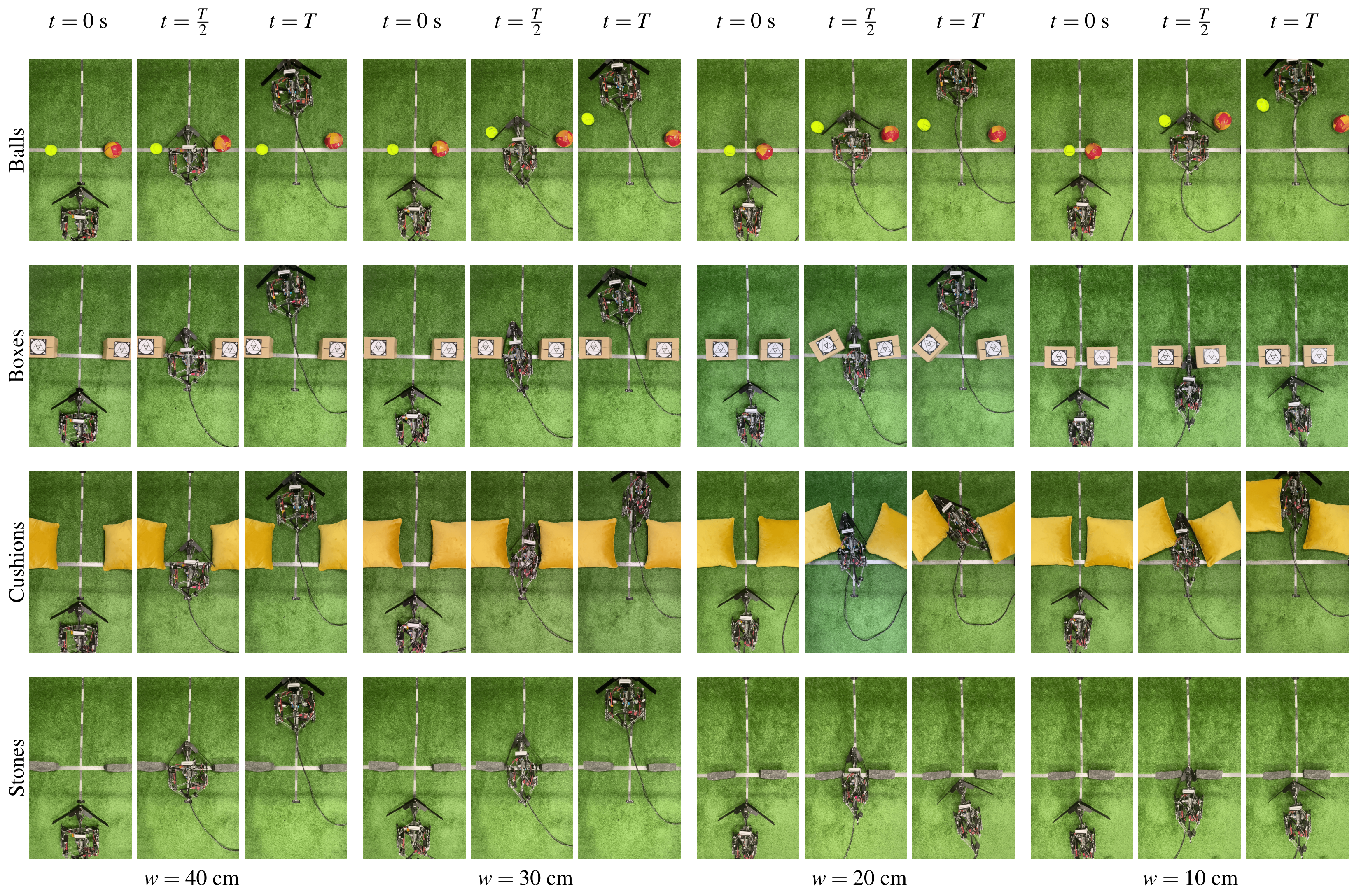

Supplementary Figure 7. Exclusively using the Obstacle Traversal Algorithm, from an approach angle of 90° the robot attempts to traverse various obstacles with different physical properties placed apart by decreasing distances. Timestamps of the still frames are shown in the column headers, where T is the time it takes for the robot to complete its navigation attempt. Obstacle names are listed in the row headers and gap widths are detailed below the figures.

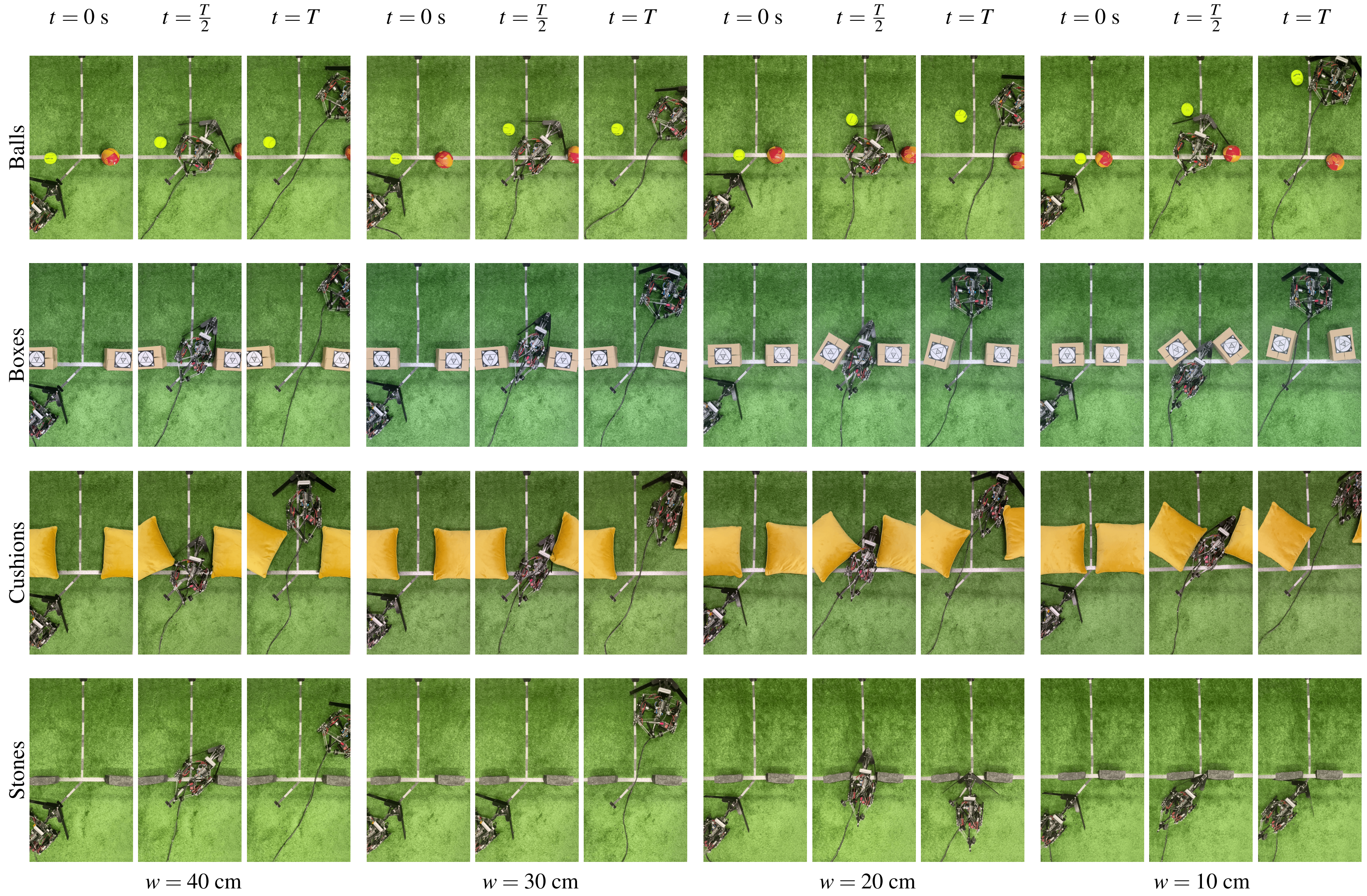

Supplementary Figure 8. Exclusively using the Obstacle Traversal Algorithm, from an approach angle of 45° the robot attempts to traverse various obstacles with different physical properties placed apart by decreasing distances. Timestamps of the still frames are shown in the column headers, where T is the time it takes for the robot to complete its navigation attempt. Obstacle names are listed in the row headers and gap widths are detailed below the figures.

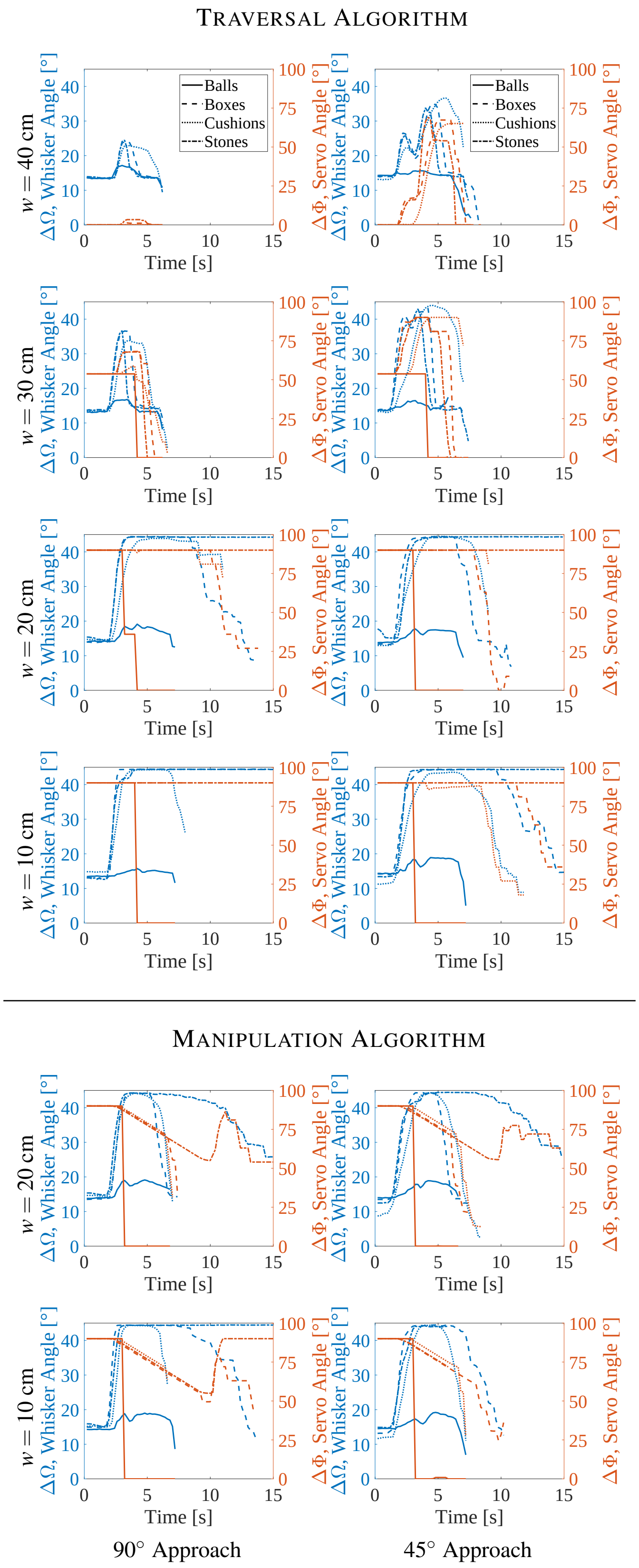

Supplementary Figure 9. Mean measurements of the whisker angle $\Delta\Omega$ (in blue) and servo angle $\Delta\Phi$ (in orange) of the robot employing the Traversal and Manipulation Algorithms for the various obstacles at different apertures approached from different angles.
